# *Ecocultural* or *Biocultural*? Towards Appropriate Terminologies in Biocultural Diversity

**DOI:** 10.3390/biology11020207

**Published:** 2022-01-28

**Authors:** F. Merlin Franco

**Affiliations:** Institute of Asian Studies, Universiti Brunei Darussalam, Gadong BE1410, Brunei; merlin.francis@ubd.edu.bn

**Keywords:** biocultural diversity, biocultural anthropology, biocultural studies, ecocultural studies, cultural studies, ecoculture, cultural diversity, environment, probabilism, environmental studies

## Abstract

**Simple Summary:**

Biocultural diversity espouses an inseparable link between biological, cultural, and linguistic diversity. Biocultural diversity is not alone in using the term ‘biocultural’. The term has been used in biocultural studies within anthropology decades ahead of biocultural studies. Both biocultural studies and biocultural diversity use the term ‘biocultural’ as adjective to generate new terminologies such as ‘biocultural approach’ with varying connotations. Such a confusing scenario might hinder theoretical advancements in biocultural diversity. Hence, I propose that proponents of biocultural diversity explore possibilities of adapting the term ‘ecoculture’ from cultural studies. Perhaps using the term ‘ecocultural’ instead of ‘biocultural’ as a descriptor to coin terminologies could solve confusions arising from the expanding usage of the term ‘bioculture’.

**Abstract:**

Biocultural diversity has made notable contributions that have furthered our understanding of the human culture-nature interrelationship. However, the usage of the term ‘biocultural’ is not unique to biocultural diversity. It was first used in biocultural studies within anthropology decades ahead of biocultural diversity. The existing literature on biocultural diversity does not acknowledge the prior existence of biocultural studies, or provide a clear demarcation between usages of the two terms. In this article, I discuss the varying contexts in usage of the term ‘biocultural’ between biocultural diversity and biocultural anthropology. While biocultural diversity deals with the linkages between biological, cultural, and linguistic diversity, biocultural studies in anthropology deal with the deterministic influence of physical and social environment on human biology and wellbeing. In biocultural studies, ‘biocultural’ refers to the integration of methodically collated cultural data with biological and environmental data. ‘Bio’ in biocultural anthropology therefore denotes biology, unlike biocultural diversity where it refers to biodiversity. Both biocultural studies and biocultural diversity apply ‘biocultural’ as descriptor to generate overlapping terminologies such as ‘biocultural approach’. Such a confusing scenario is not in the interest of biocultural diversity, as it would impede theoretical advancements. I propose that advocates of biocultural diversity explore its harmonies with ecoculturalism and the possibilities of suitably adapting the term ‘ecoculture’ in lieu of ‘bioculture’. Using ‘ecocultural’ instead of ‘biocultural’ as a descriptor to coin terminologies could solve confusions arising from the expanding usage of the term ‘bioculture’.

## 1. Introduction

Biocultural diversity is the diversity of life in all of its manifestations: biological, cultural, and linguistic, which are interrelated (and possibly coevolved) within a complex socio-ecological adaptive system [1] (p. 269). The origin of the concept of biocultural diversity could be traced back to 1988, when ‘The Declaration of Belém’ from the First International Congress of Ethnobiology recognized an ‘inextricable link’ between biological and cultural diversity [2]. By recognizing cultural diversity, biocultural diversity offers a better approach to understand the interrelationships between humans and nature [3]. In recent years, there has also been an emphasis on the dynamic, reciprocal nature of the human culture-nature relationship [4]. The accordance of prominence to cultural and linguistic diversities distinguishes biocultural diversity from biodiversity (or biological diversity) defined as the “variety and variability among living organisms and the ecological complexes in which they occur” [5] (see also [6,7]). Biodiversity with its species conservation-oriented approach excluded local people and their interrelationship with nature. Anthropologists helped in bridging the gap between biodiversity conservation and local communities [8], while the definition of biodiversity also evolved to accommodate cultural diversity [9]. However, biodiversity falls short of the holistic approach towards the human culture-language-biodiversity complex advocated by biocultural diversity. The number of publications using the biocultural diversity framework has been growing since the 2000s [3,10]. As outlined by Luisa Maffi [3], these studies tend to have four major foci: (i) the relationship between language, traditional knowledge, and the environment; (ii) common threats to biological, cultural, and linguistic diversities; (iii) conservation and revitalization of biocultural diversity; and (iv) biocultural diversity and human rights. 

Maffi describes biocultural diversity as a ‘multifaceted field of research’ in her landmark publications that provide the theoretical underpinnings of this area of study [1,3]. Some academics (especially ethnobiologists) approach it as a conceptual framework that bridges the nature-culture divide [11]. However, biocultural diversity is not alone in using the term ‘biocultural’. The term ‘biocultural’ was first used in biocultural studies within anthropology, whose origin can be traced to the 1960s (decades ahead of biocultural diversity) [12,13,14]. Biocultural studies in anthropology deal with the influence of biological and cultural environment on human biology and wellbeing [15]. Biocultural diversity on the other hand, deals with the linkages between biological, cultural, and linguistic diversities. Although biocultural diversity has accumulated a significant volume of literature since the 2000s [10], the current literature on biocultural diversity does not acknowledge the prior existence of biocultural studies in anthropology or provide a clear demarcation between the two concepts. Maffi’s article published in the Annual Review of Anthropology in 2005 [3], a reputed journal in the field of anthropology, misses the opportunity to provide a clear demarcation between the two paradigms. Such a scenario creates confusion in the minds of young anthropologists and ethnobiologists getting acquainted with the term ‘biocultural’. Clarity at the level of definitions and usage of appropriate terminologies are quintessential for theoretical advancements in any field of enquiry [16]. Therefore, in this article, I discuss the different contexts in usage of the term ‘biocultural’ as a descriptor in biocultural diversity and biocultural studies within anthropology. I highlight the confusion caused by the usage of the same term in diverging contexts by both the paradigms. Lastly, I conclude by proposing that the term ‘ecocultural’ is a better alternative to use as a descriptor in studies using the biocultural diversity framework.

## 2. Biocultural Studies in Anthropology

The origins of biocultural studies within anthropology could be traced to 1930s when anthropologists including W. Montague Cobb studied the influence of social environments on human health [17]. In the 1960s, specific research methods in biocultural studies were advanced by the International Biological Program [18]. However, specific usage of the term only appears in the 1970s [19]. There are considerable variations in the conceptualization of biocultural studies, with some anthropologists nesting it within biological anthropology and others explicitly advocating biocultural anthropology as a sub-discipline of biological anthropology [12,19,20]. Given the variations in conceptualizations, there have been calls to focus on what biocultural studies does in the contemporary world, rather than its definition [21]. Prior to 1998, biocultural approaches in anthropology aimed to understand the deterministic pathways by which social, economic, cultural, and ecological factors influence human biology and wellbeing [12,13,15]. Biocultural studies has evolved since then to emphasize reciprocity in human-environment relationship, with the conceptualization of the field expanded to encompass human niche construction [21]. However, the deterministic influence of biological and cultural factors on human biology and wellbeing, especially the study of human physiological and cultural adaptation to environmental conditions [20], continues to be the major focus of the field [22]. Biocultural studies within the realm of biological anthropology and associated fields see humans as ‘biological, social, and cultural beings’ [12], and the discipline has strong synergies to medical anthropology, ecological anthropology, and political economy [21,22]. A small number of studies also tend to employ evolutionary theory or political economic analyses [19]. Various associations of anthropology have organized conferences in biocultural studies/anthropology, and a plethora of articles have also been published in leading journals in anthropology [19,23].

### ‘Biocultural’ in Biocultural Studies

The coining of the term ‘biocultural’ is believed to be the result of anthropology’s quest for turning into a holistic field [19,24]. According to Ann McElroy [12], the ideal sense of ‘biocultural’ in biocultural studies is the integration of methodically collated cultural data with biological and environmental data. The ‘bio’ in biocultural studies therefore denotes biology. There appears to be little consensus on the usage of the term within biocultural studies, or a theoretical framework that outlines the constitutive elements and processes. In their bibliometric review, Wiley and Cullin found tremendous variation in its usage [19]. In the majority of studies, the term implies the influence of social environment on human biology. However, there were 180 terms formed using ‘biocultural’ as a descriptor (adjective) often with little clarity on what they intend to convey. In Table 1, I provide subjective examples of variations in direct usage of the term ‘biocultural’ in biocultural studies. In these examples, ‘biocultural’ has been combined with terms such as ‘adaptation’ and ‘approach’ to produce varying connotations. Notable usage includes ‘biocultural diversity/variations’ to denote morphological variations in human populations, induced by cultural practices such as intentional body modification practices [25].

## 3. ‘Biocultural’ in Biocultural Diversity: Similar Terminologies, but Confounding Usage

Biocultural diversity deals with the inextricable linkages between biological, cultural, and linguistic diversities [1,3,37,38]. It thus focuses on the manifestation of these diversities, and not the influence of cultural and social environment on human biology advocated by biocultural studies [12]. The ‘bio’ in biocultural diversity therefore refers to biodiversity and not biology as in biocultural studies. According to Mercon et al. [39], the term ‘bioculture’ is employed in biocultural diversity “to emphasize tightly intertwined and co-evolving social-ecological systems, cultural dimensions and implications in such systems”. A subjective scan of published academic literature shows an overlap in terminologies coined by applying the term ‘biocultural’ as descriptor between both the paradigms, with the context of its usage in biocultural diversity being different from biocultural studies in anthropology (Table 1 and Table 2). A ‘biocultural approach’ in the latter could mean understanding the influence of environment on human health by examining parameters such as obesity and nutritional status [29], while in biocultural diversity it would mean an approach that recognizes the co-existence of biological and cultural diversity, and the linkages between them [39,40]. If biocultural conservation in biocultural diversity means to conserve biological and cultural diversity [41], in the context of biocultural studies it would mean conserving the diverse patterns of environmental influence on human wellbeing when applied. Thus, the possibilities to generate various terminologies incorporating ‘biocultural’ in both biocultural studies and biocultural diversity are innumerable, leading to a confusing scenario, a complexity that would only grow from here unless resolved. Such a confusing scenario is further exemplified by the usage of ‘biocultural diversity’ as such in biocultural studies to refer to morphological variations in human populations [25]. 

Of the articles published in AAA journals during the 2000–2014 period, Wiley and Cullin found 3% to use the term ‘biocultural’ [19]. These usages are in a context different from that of biocultural diversity, except for those explicitly dealing with biocultural diversity. Although the authors recognize that biocultural diversity is distinct from biocultural studies/anthropology, they call upon academics to explore ways to harmonize these two paradigms. However, the differences between biocultural studies in anthropology and biocultural diversity are vast to reconcile. If biocultural studies in anthropology are concerned with the influence of biological and cultural factors on human biology and health, then biocultural diversity is about “the living network made up of the millions of species and animals and the thousands of human culture and languages that have evolved on earth” [59]. The conceptualization of biocultural studies in anthropology has been expanded recently to emphasize the reciprocal relationship between humans and nature [21], marking a conscientious shift from environmental determinism to environmental probabilism (See: Lewthwaite [60]). However, it is undeniable that the core area of focus has been human health and wellbeing, especially the deterministic impact of social and physical environment on human health [19,22]. Contrarily, from its inception, biocultural diversity has recognized the ever-evolving complex and reciprocal interaction between nature and humans, thus assuming a probabilistic stand [1,11]. Furthermore, biocultural diversity does not focus on the influence of the physical and biological environments on human biology and health. 

The evident divergence in conceptualization of ‘biocultural’, and its confounding usages in biocultural studies and biocultural diversity is unaffordable in academia. In an era where keywords increasingly play an important role in access to knowledge [61], young anthropologists, ethnobiologists, linguists, or geographers undertaking literature review would invariably be confused in the diverse usages. Of the 199 articles tagged with ‘biocultural’ in the Scopus database for the year 2020 (Supplementary Materials File S1), the majority (*n* = 122) have used the biocultural diversity framework, while the remaining were from biocultural studies. Although this indicates the increasing popularity and acceptability of biocultural diversity among researchers probing the human-nature nexus, it also points out the confusing scenario in the usage of ‘bioculture/biocultural’. Indeed, as the younger of the two paradigms, the onus is on biocultural diversity to differentiate itself from biocultural studies. A radical step here is to debate the possibility of re-branding biocultural diversity as ecocultural diversity. However, this would require serious effort in building consensus among advocates of biocultural diversity. A more acceptable middle-path could be to retain biocultural diversity as such at the conceptual level but to use ‘ecocultural’ instead of ‘biocultural’ as descriptor. Thus, the term ‘biocultural approach’ in biocultural diversity would become ‘ecocultural approach’ instead, ‘biocultural revitalization’ would become ‘ecocultural revitalization’, and so on [62,63]. 

## 4. The Need for Considering ‘Ecoculture’ in Biocultural Diversity

The term ‘ecoculture’ is popular in cultural studies, environmental communication, and psychology where it signifies the reciprocal and inseparable link between ecology and culture [62,64,65,66], a paradigm referred to as ‘ecoculturalism’ [67]. An ecoculturalist perspective advocates that sociocultural identity is inseparable from ecology [68]. It also recognizes that local knowledge and memories of the dynamic link between the non-human component of landscapes and human culture shape ecocultural identities and promote resilience [69,70,71]. In tourism, ecoculturalism offers an opportunity to appreciate both the cultural and ecological aspects of destinations [72]. Like biocultural diversity, ecoculturalism also recognizes that ecological crises leads to cultural crises [70]. Meanwhile in cultural studies, it has also been hotly debated if the term ecoculturalism should be abandoned, as the field of cultural studies addresses the nature-culture dualism adequately [67,73]. Bohm et al. [74] refer to those communities living an ecocultural lifestyle that recognizes, demonstrates, and nurtures the deep linkages between social and ecological environments as ‘ecocultures’—an application that is similar to ‘biocultures’ in biocultural diversity [56] but different from ‘biocultures’ as used in biocultural studies [35,36,75]. Ecocultures or ecocultural communities appreciate the reciprocal relationship between nature and culture, the need for nurturing ecosystem health, recognition of all lifeforms as sentient beings, and the importance of a healthy nature-culture relationship [76,77,78,79]. These communities are characterized by ethical principles that prioritize the nurturing of sociological and ecological wellbeing, recognize that wellbeing does not constitute of economic wellbeing alone, consider humans as a part of nature, and strive to conserve and sustain natural, human and social capitals [74,80]. The usage of the term ‘ecocultural’ in cultural studies, psychology and elsewhere, and ‘biocultural’ in biocultural diversity are remarkably similar as they both recognize the inextricable link between ecosystems and culture [37]. Given that the term ‘biocultural’ has been used in a different context in biocultural studies within anthropology long before the birth of biocultural diversity, it is in the interest of the latter to suitably adapt the term ‘ecoculture’ to distinguish its program from that of biocultural studies in anthropology.

## 5. Conclusions

Biocultural studies within anthropology originated decades ahead of biocultural diversity. Modern conceptualizations of biocultural studies have expanded its scope to include reciprocity in human-environment relationships. Yet, the deterministic influence of physical and social environment on human health and wellbeing continues to be the major focus of the field. ‘Bio’ in biocultural studies refers to human biology, while ‘biocultural’ refers to the integration of methodically collated cultural data with biological and environmental data. In biocultural diversity, ‘bio’ refers to biodiversity, and ‘biocultural’ refers to the co-evolving biological and cultural diversity and the linkages between them. Biocultural diversity is a well-defined paradigm with a robust theoretical framework. From its inception, the paradigm has espoused a probabilistic relationship between biological, linguistic, and cultural diversity. However, the usage of ‘biocultural’ in a context differing from that of biocultural studies has led to a confusing scenario with overlapping terminologies such as ‘biocultural approach’, coined in biocultural studies, and biocultural diversity, with varying connotations. The scenario could become more confounding in the future, with the emergence of the usage of ‘biocultural diversity’ as such in biocultural studies in a context other than that of biocultural diversity. Being the younger of the two paradigms, the onus is on biocultural diversity to demarcate itself from biocultural studies and steer itself clear of confusing terminologies. I propose that advocates of biocultural diversity explore its harmonies with ecoculturalism and the possibilities of suitably adapting the term ‘ecoculture’ in lieu of ‘biocultural’. Using ‘ecocultural’ instead of ‘biocultural’ as descriptor to coin terminologies could solve much of the confusions arising from the expanding usage of the term ‘biocultural’.

## Figures and Tables

**Table 1:** Examples of terms coined using ‘biocultural’ as descriptor in biocultural studies.

Terminology	Usage
Biocultural adaptation	Influence of environment and lifestyle on human physiology [26,27].
Biocultural analyses/perspectives	Linking culture and biology to unravel how biological phenomena such as birth are affected by cultural interpretations and practices [28].
Biocultural approach	Environment influencing obesity and nutritional status [29]. “Humans as biological, social and cultural beings in relation to the environment” McElroy [12] cited in Khongsdier [30].
Biocultural diversity/variations	Human morphological variations induced by diverse range of intentional body modification practices [25].
Biocultural evolution	Evolution of biological and cultural characteristics [31].
Biocultural model	A model that could be useful in “conceptualizing the complex interaction of biological, cultural and psychosocial factors in the process of human pain perception” [32].
Biocultural predictors	Combination of biological and cultural factors [33].
Bio-cultural sciences	“Bio-cultural sciences highlight the notion that human behaviour is the joint and co-constructive expression of biological–genetic and cultural–societal processes and conditions.” [34]
Biocultural studies	“Questions of human biology and medical ecology that specifically include social, cultural, or behavioural variables in the research design” [12].
Biocultures	Re-thinking of culture and history by considering their ‘inextricable’ relationship with biology [35]. “Cultural spheres where biomedicine extends beyond the formal institutions of the clinic, the hospital, the lab, and so forth and is incorporated into broader social practices and rationalities” [36].

Note: For a comprehensive list of descriptors published between 2000 and 2014, see Wiley and Cullin [19].

**Table 2:** Examples of terms coined using ‘biocultural’ as descriptor in biocultural diversity.

Terminology	Usage
Biocultural approach	Recognising human beings and non-humans as co-habitants of ecosystems [42,43]. “Biocultural approaches are an emergent area of study that conceptualize interrelationships between cultures and the environment” [40].
Biocultural approaches to conservation	“Conservation actions made in the service of sustaining the biophysical and sociocultural components of dynamic, interacting, and interdependent social–ecological systems” [41]
Biocultural characteristics	Undefined [44].
Biocultural conservation	Stemming the dual loss of biological and cultural diversity [41].
Biocultural design	“People are creative agents with knowledge, values and skills that allow them to shape their everyday lives” [45]
Biocultural ethics	“Recovering the vital links between biological and cultural diversity, between the habits and the habitats of the inhabitants” [46].
Biocultural heritage	Biodiversity and culture as heritage [47].
Biocultural homogenization	“Simultaneous and interdigitated losses of native biological and cultural diversity at local, regional, and global scales” [46].
Biocultural importance	Biological and cultural importance of plants, animals and landscapes [48,49].
Biocultural indicators	Foreseeable seasonal events such as flowering of calendar plants that are culturally significant to local communities [50].
Biocultural interactions	Interactions between local people and ecosystems [51].
Biocultural landscape	Landscapes that integrate “economic, social, cultural and environmental processes in time and space” [52].
Biocultural learning	“Learning complexity in and about nature, particularly to the dimensions and processes involved when people have nature as a workplace” [53].
Biocultural memory	“The human memory is the result of interactions between biological and cultural traits, considered as biocultural memory” [54].
Biocultural refugia/Bio-cultural refugia	“Physical places that not only shelter farm biodiversity, but also carry knowledge and experiences about practical management of how to produce food while stewarding biodiversity and ecosystem services” [55].
Biocultural systems	Systems moulded jointly by biological and cultural forces [38].
Biocultures	“A bioculture is a local collection of humans, other species, and their interactions” [56]
Collective biocultural heritage	“Knowledge, innovations and practices of indigenous and local communities which are collectively held and inextricably linked to traditional resources and territories, local economies, the diversity of genes, varieties, species and ecosystems, cultural and spiritual values, and customary laws shaped within the socio-ecological context of communities” [57]
Indigenous biocultural Knowledge	“Knowledge that encompasses people, language and culture and their relationship to the environment” [58]

## Data Availability

Data supporting reported results can be found in the Supplementary File S1.

## Supplementary Materials

The following are available online at https://www.mdpi.com/article/10.3390/biology11020207/s1. File S1: Publications tagged with ‘biocultural’ in the Scopus database for the year 2020.
