# Supplementary material for: Ecocultural or Biocultural? Towards Appropriate Terminologies in Biocultural Diversity
Source: Biology (Basel). 2022 Jan 28;11(2):207. doi: 10.3390/biology11020207 (PMC8869769; doi:10.3390/biology11020207)
Supplement: Supplementary file 1 [file biology-11-00207-s001.zip › biology-1508030 FileS1 - Scopus 2020.pdf]

Supplementary Materials File S1

Publications tagged with 'biocultural' in the Scopus database for the year 2020.

Export date: 27 Sep 2021

Total No. of Publications: 199

Publications relevant to the biocultural diversity framework: 112 (highlighted in green font)

1. Hanspach, J., Jamila Haider, L., Oteros-Rozas, E., Stahl Olafsson, A., Gulsrud, N.M., Raymond, C.M., Torralba, M., Martín-López, B., Bieling, C., García-Martín, M., Albert, C., Beery, T.H., Fagerholm, N., Díaz-Reviriego, I., Drews-Shambroom, A., Plieninger, T. Biocultural approaches to sustainability: A systematic review of the scientific literature (2020) People and Nature, 2 (3), pp. 643-659. DOI: 10.1002/pan3.10120

2. Schuster, R.C., Butler, M.S., Wutich, A., Miller, J.D., Young, S.L., Household Water Insecurity Experiences-Research Coordination Network (HWISE-RCN) "If there is no water, we cannot feed our children": The far-reaching consequences of water insecurity on infant feeding practices and infant health across 16 low- and middle-income countries (2020) American Journal of Human Biology, 32 (1), art. no. e23357,DOI: 10.1002/ajhb.23357

3. McElwee, P., Fernández-Llamazares, Á., Aumeeruddy-Thomas, Y., Babai, D., Bates, P., Galvin, K., Guèze, M., Liu, J., Molnár, Z., Ngo, H.T., Reyes-García, V., Roy Chowdhury, R., Samakov, A., Shrestha, U.B., Díaz, S., Brondízio, E.S. Working with Indigenous and local knowledge (ILK) in large-scale ecological assessments: Reviewing the experience of the IPBES Global Assessment (2020) Journal of Applied Ecology, 57 (9), pp. 1666-1676. DOI: 10.1111/1365-2664.13705

4. Columbu, A., Chiarini, V., Spötl, C., Benazzi, S., Hellstrom, J., Cheng, H., De Waele, J. Speleothem record attests to stable environmental conditions during Neanderthal–modern human turnover in southern Italy (2020) Nature Ecology and Evolution, 4 (9), pp. 1188-1195. DOI: 10.1038/s41559-020-1243-1

5. Borelli, T., Hunter, D., Padulosi, S., Amaya, N., Meldrum, G., de Oliveira Beltrame, D.M., Samarasinghe, G., Wasike, V.W., Güner, B., Tan, A., Dembélé, Y.K., Lochetti, G., Sidibé, A., Tartanac, F. Local solutions for sustainable food systems: The contribution of orphan crops and wild edible species (2020) Agronomy, 10 (2), art. no. 231, DOI: 10.3390/agronomy10020231

6. Wutich, A. Water insecurity: An agenda for research and call to action for human biology (2020) American Journal of Human Biology, 32 (1), art. no. e23345. DOI: 10.1002/ajhb.23345

7. Kalle, R., Belichenko, O., Kuznetsova, N., Kolosova, V., Prakofjewa, J., Stryamets, N., Mattalia, G., Šarka, P., Simanova, A., Prūse, B., Mezaka, I., Sõukand, R. Gaining momentum: Popularization of Epilobium angustifolium as food and recreational tea on the Eastern edge of Europe (2020) Appetite, 150, art. no. 104638. DOI: 10.1016/j.appet.2020.104638

8. Leatherman, T., Goodman, A. Building on the biocultural syntheses: 20 years and still expanding (2020) American Journal of Human Biology, 32 (4), art. no. e23360. DOI: 10.1002/ajhb.23360

9. Searle, A., Turnbull, J. Resurgent natures? More-than-human perspectives on COVID-19 (2020) Dialogues in Human Geography, 10 (2), pp. 291-295. DOI: 10.1177/2043820620933859

10. Winter, K.B., Lincoln, N.K., Berkes, F., Alegado, R.A., Kurashima, N., Frank, K.L., Pascua, P., Rii, Y.M., Reppun, F., Knapp, I.S.S., McClatchey, W.C., Ticktin, T., Smith, C., Franklin, E.C., Oleson, K., Price, M.R., McManus, M.A., Donahue, M.J., Rodgers, K.S., Bowen, B.W., Nelson, C.E., Thomas, B., Leong, J.-A., Madin, E.M.P., Rivera, M.A.J., Falinski, K.A., Bremer, L.L., Deenik, J.L., Gon, S.M., III, Neilson, B., Okano, R., Olegario, A., Nyberg, B., Hiʻilei Kawelo, A., Kotubetey, K., Kānekoaā Kukea-Shultz, J., Toonen, R.J. Ecomimicry in indigenous resource management: Optimizing ecosystem services to achieve resource abundance, with examples from Hawaiʻi (2020) Ecology and Society, 25 (2), art. no. 26, pp. 1-18. DOI: 10.5751/ES-11539-250226

11. Blaikie, C. Critically endangered? Medicinal plant cultivation and the reconfiguration of Sowa rigpa in ladakh (2020) Asian Medicine, 5 (2), pp. 243-272. DOI: 10.1163/157342109X568801

12. Bogin, B., Varea, C. COVID-19, crisis, and emotional stress: A biocultural perspective of their impact on growth and development for the next generation (2020) American Journal of Human Biology, 32 (5), art. no. e23474. DOI: 10.1002/ajhb.23474

13. Constant, N.L., Taylor, P.J. Restoring the forest revives our culture: Ecosystem services and values for ecological restoration across the rural-urban nexus in South Africa (2020) Forest Policy and Economics, 118, art. no. 102222. DOI: 10.1016/j.forpol.2020.102222

14. Albert, J., Bogard, J., Siota, F., McCarter, J., Diatalau, S., Maelaua, J., Brewer, T., Andrew, N. Malnutrition in rural Solomon Islands: An analysis of the problem and its drivers (2020) Maternal and Child Nutrition, 16 (2), art. no. e12921. DOI: 10.1111/mcn.12921

15. Vierikko, K., Gonçalves, P., Haase, D., Elands, B., Ioja, C., Jaatsi, M., Pieniniemi, M., Lindgren, J., Grilo, F., Santos-Reis, M., Niemelä, J., Yli-Pelkonen, V. Biocultural diversity (BCD) in European cities – Interactions between motivations, experiences and environment in public parks (2020) Urban Forestry and Urban Greening, 48, art. no. 126501. DOI: 10.1016/j.ufug.2019.126501

16. Kalle, R., Sõukand, R., Pieroni, A. Devil is in the details: Use of wild food plants in historical Võromaa and Setomaa, present-day Estonia (2020) Foods, 9 (5), art. no. 570. DOI: 10.3390/foods9050570

17. Fischer, L.K., Kowarik, I. Connecting people to biodiversity in cities of tomorrow: Is urban foraging a powerful tool? (2020) Ecological Indicators, 112, art. no. 106087. DOI: 10.1016/j.ecolind.2020.106087

18. Yang, J., Chen, W.-Y., Fu, Y., Yang, T., Luo, X.-D., Wang, Y.-H., Wang, Y.-H. Medicinal and edible plants used by the Lhoba people in Medog County, Tibet, China (2020) Journal of Ethnopharmacology, 249, art. no. 112430. DOI: 10.1016/j.jep.2019.112430

19. McKemey, M., Ens, E., Rangers, Y.M., Costello, O., Reid, N. Indigenous knowledge and seasonal calendar inform adaptive savanna burning in northern Australia (2020) Sustainability (Switzerland), 12 (3), pp. 1-18. DOI: 10.3390/su12030995

20. Sarmiento, F.O. Montology manifesto: echoes towards a transdisciplinary science of mountains (2020) Journal of Mountain Science, 17 (10), pp. 2512-2527. DOI: 10.1007/s11629-019-5536-2

21. Mattalia, G., Sõukand, R., Corvo, P., Pieroni, A. Wild food thistle gathering and pastoralism: An inextricable link in the biocultural landscape of Barbagia, central Sardinia (Italy) (2020) Sustainability (Switzerland), 12 (12), art. no. 5105. DOI: 10.3390/su12125105

22. Aziz, M.A., Abbasi, A.M., Ullah, Z., Pieroni, A. Shared but threatened: The heritage of wild food plant gathering among different linguistic and religious groups in the Ishkoman and Yasin Valleys, North Pakistan (2020) Foods, 9 (5), art. no. 601. DOI: 10.3390/foods9050601

23. Aziz, M.A., Ullah, Z., Pieroni, A. Wild food plant gathering among kalasha, yidgha, nuristani and khowar speakers in chitral, NW Pakistan (2020) Sustainability (Switzerland), 12 (21), art. no. 9176, pp. 1-23. DOI: 10.3390/su12219176

24. Fernández-Llamazares, Á., Terraube, J., Gavin, M.C., Pyhälä, A., Siani, S.M.O., Cabeza, M., Brondizio, E.S. Reframing the Wilderness Concept can Bolster Collaborative Conservation (2020) Trends in Ecology and Evolution, 35 (9), pp. 750-753. DOI: 10.1016/j.tree.2020.06.005

25. Mattalia, G., Stryamets, N., Pieroni, A., Sõukand, R. Knowledge transmission patterns at the border: Ethnobotany of Hutsuls living in the Carpathian Mountains of Bukovina (SW Ukraine and NE Romania) (2020) Journal of Ethnobiology and Ethnomedicine, 16 (1), art. no. 391. DOI: 10.1186/s13002-020-00391-3

26. Hoke, M.K., Schell, L.M. Doing biocultural anthropology: Continuity and change (2020) American Journal of Human Biology, 32 (4), art. no. e23471. DOI: 10.1002/ajhb.23471

27. Gallois, S., Heger, T., van Andel, T., Sonké, B., Henry, A.G. From Bush Mangoes to Bouillon Cubes: Wild Plants and Diet among the Baka, Forager-Horticulturalists from Southeast Cameroon (2020) Economic Botany, 74 (1), pp. 46-58. DOI: 10.1007/s12231-020-09489-x

28. Nieves-Colón, M.A., Pestle, W.J., Reynolds, A.W., Llamas, B., De La Fuente, C., Fowler, K., Skerry, K.M., Crespo-Torres, E., Bustamante, C.D., Stone, A.C., Mulligan, C. Ancient DNA Reconstructs the Genetic Legacies of Precontact Puerto Rico Communities (2020) Molecular Biology and Evolution, 37 (3), pp. 611-626. DOI: 10.1093/molbev/msz267

29. Winter, K.B., Ticktin, T., Quazi, S.A. Biocultural restoration in Hawai‘i also achieves core conservation goals (2020) Ecology and Society, 25 (1), art. no. 26. DOI: 10.5751/ES-11388-250126

30. MacPherson, E., Ventura, J.T., Ospina, F.C. Constitutional law, ecosystems, and indigenous peoples in colombia: Biocultural rights and legal subjects (2020) Transnational Environmental Law, 9 (3), pp. 521-540. DOI: 10.1017/S204710252000014X

31. Haider, L.J., Boonstra, W.J., Akobirshoeva, A., Schlüter, M. Effects of development interventions on biocultural diversity: a case study from the Pamir Mountains (2020) Agriculture and Human Values, 37 (3), pp. 683-697. DOI: 10.1007/s10460-019-10005-8

32. Brewis, A.A., Piperata, B., Thompson, A.L., Wutich, A. Localizing resource insecurities: A biocultural perspective on water and wellbeing (2020) Wiley Interdisciplinary Reviews: Water, 7 (4), art. no. e1440. DOI: 10.1002/wat2.1440

33. Grove, R., Pim, J.E., Serrano, M., Cidrás, D., Viles, H., Sanmartín, P. Pastoral stone enclosures as biological cultural heritage: Galician and cornish examples of community conservation (2020) Land, 9 (1), art. no. 9. DOI: 10.3390/land9010009

34. Laffranchi, Z., Charisi, D., Jiménez-Brobeil, S.A., Milella, M. Gendered division of labor in a Celtic community? A comparison of sex differences in entheseal changes and long bone shape and robusticity in the pre-Roman population of Verona (Italy, third–first century BC) (2020) American Journal of Physical Anthropology, 173 (3), pp. 568-588. DOI: 10.1002/ajpa.24111

35. Nelson, R.G. Beyond the Household: Caribbean Families and Biocultural Models of Alloparenting (2020) Annual Review of Anthropology, 49, pp. 355-372. DOI: 10.1146/annurev-anthro-102218-011140

36. Rayne, A., Byrnes, G., Collier-Robinson, L., Hollows, J., McIntosh, A., Ramsden, M., Kāti Huikai, Kāi Tahu, Rupene, M., Ngāi Tūāhuriri, Ngāi Tahu, Tamati-Elliffe, P., Thoms, C., Ngāti Kurī, Ngāi Tahu, Steeves, T.E. Centring Indigenous knowledge systems to re-imagine conservation translocations (2020) People and Nature, 2 (3), pp. 512-526. DOI: 10.1002/pan3.10126

37. Herse, M.R., Lyver, P.O., Scott, N., Mcintosh, A.R., Coats, S.C., Gormley, A.M., Tylianakis, J.M. Engaging Indigenous Peoples and Local Communities in Environmental Management Could Alleviate Scale Mismatches in Social-Ecological Systems (2020) BioScience, 70 (8), pp. 699-707. DOI: 10.1093/biosci/biaa066

38. Avilez-López, T., Van Der Wal, H., Aldasoro-Maya, E.M., Rodríguez-Robles, U. Home gardens' agrobiodiversity and owners' knowledge of their ecological, economic and socio-cultural multifunctionality: A case study in the lowlands of Tabasco, México (2020) Journal of Ethnobiology and Ethnomedicine, 16 (1), art. no. 42. DOI: 10.1186/s13002-020-00392-2

39. Dressler, W.W. The construction of the cultural niche: A biocultural model (2020) American Journal of Human Biology, 32 (4), art. no. e23311. DOI: 10.1002/ajhb.23311

40. Blakey, M.L. On the biodeterministic imagination (2020) Archaeological Dialogues, 27 (1), pp. 1-16. DOI: 10.1017/S1380203820000021

41. Fernández-Llamazares, Á., Virtanen, P.K. Game masters and Amazonian Indigenous views on sustainability (2020) Current Opinion in Environmental Sustainability, 43, pp. 21-27. DOI: 10.1016/j.cosust.2020.01.004

42. Mattalia, G., Sõukand, R., Corvo, P., Pieroni, A. Dissymmetry at the Border: Wild Food and Medicinal Ethnobotany of Slovenes and Friulians in NE Italy (2020) Economic Botany, 74 (1). DOI: 10.1007/s12231-020-09488-y

43. Franco, F.M., Chaw, L.L., Bakar, N., Abas, S.N.H. Socialising over fruits and vegetables: The biocultural importance of an open-air market in Bandar Seri Begawan, Brunei Darussalam (2020) Journal of Ethnobiology and Ethnomedicine, 16 (1), art. no. 6. DOI: 10.1186/s13002-020-0356-6

44. Bogadóttir, R. The social metabolism of quiet sustainability in the faroe Islands (2020) Sustainability (Switzerland), 12 (2), art. no. 735. DOI: 10.3390/su12020735

45. Schell, L.M. Modern water: A biocultural approach to water pollution at the Akwesasne Mohawk Nation (2020) American Journal of Human Biology, 32 (1), art. no. e23348. DOI: 10.1002/ajhb.23348

46. Kohrt, B.A., Ottman, K., Panter-Brick, C., Konner, M., Patel, V.Why we heal: The evolution of psychological healing and implications for global mental health (2020) Clinical Psychology Review, 82, art. no. 101920. DOI: 10.1016/j.cpr.2020.101920

47. Singh, R.K., Kumar, A., Singh, A., Singhal, P. Evidence that cultural food practices of Adi women in Arunachal Pradesh, India, improve social-ecological resilience: insights for Sustainable Development Goals (2020) Ecological Processes, 9 (1), art. no. 29. DOI: 10.1186/s13717-020-00232-x

48. Russo, G., Beritognolo, I., Bufacchi, M., Stanzione, V., Pisanelli, A., Ciolfi, M., Lauteri, M., Brush, S.B. Advances in biocultural geography of olive tree (Olea europaea L.) landscapes by merging biological and historical assays (2020) Scientific Reports, 10 (1), art. no. 7673. DOI: 10.1038/s41598-020-64063-8

49. Reyes-González, A., Camou-Guerrero, A., del-Val, E., Ramírez, M.I., Porter-Bolland, L. Biocultural Diversity Loss: the Decline of Native Stingless Bees (Apidae: Meliponini) and Local Ecological Knowledge in Michoacán, Western México (2020) Human Ecology, 48 (4), pp. 411-422. DOI: 10.1007/s10745-020-00167-z

50. Willman, J.C., Hernando, R., Matu, M., Crevecoeur, I. Biocultural diversity in Late Pleistocene/Early Holocene Africa: Olduvai Hominid 1 (Tanzania) biological affinity and intentional body modification (2020) American Journal of Physical Anthropology, 172 (4), pp. 664-681. DOI: 10.1002/ajpa.24007

51. Hoke, M.K. A biocultural examination of home food production and child growth in highland Peru (2020) American Journal of Human Biology, 32 (4), art. no. e23438. DOI: 10.1002/ajhb.23438

52. Brewis, A., Wutich, A. Stigma: A biocultural proposal for integrating evolutionary and political-economic approaches (2020) American Journal of Human Biology, 32 (4), art. no. e23290. DOI: 10.1002/ajhb.23290

53. Tremblay, R., Landry-Cuerrier, M., Humphries, M.M. Culture and the social-ecology of local food use by indigenous communities in northern North America (2020) Ecology and Society, 25 (2), art. no. 8, pp. 1-26. DOI: 10.5751/ES-11542-250208

54. Rendón-Sandoval, F.J., Casas, A., Moreno-Calles, A.I., Torres-García, I., García-Frapolli, E. Traditional agroforestry systems and conservation of native plant diversity of seasonally dry tropical forests (2020) Sustainability (Switzerland), 12 (11), art. no. 4600. DOI: 10.3390/su12114600

55. Dori, I., Varalli, A., Seghi, F., Moggi-Cecchi, J., Sparacello, V.S. Environmental correlates of growth patterns in Neolithic Liguria (northwestern Italy) (2020) International Journal of Paleopathology, 28, pp. 112-122. DOI: 10.1016/j.ijpp.2019.12.002

56. Ojeda-Linares, C.I., Vallejo, M., Lappe-Oliveras, P., Casas, A. Traditional management of microorganisms in fermented beverages from cactus fruits in Mexico: An ethnobiological approach (2020) Journal of Ethnobiology and Ethnomedicine, 16 (1), art. no. 1. DOI: 10.1186/s13002-019-0351-y

57. Cepon-Robins, T.J., Gildner, T.E. Old friends meet a new foe (2020) Evolution, Medicine and Public Health, 2020 (1), pp. 234-248. DOI: 10.1093/EMPH/EOAA037

58. Chinnaswamy, S. SARS-CoV-2 infection in India bucks the trend: Trained innate immunity? (2020) American Journal of Human Biology. DOI: 10.1002/ajhb.23504

59. Ungar, M., McRuer, J., Liu, X., Theron, L., Blais, D., Schnurr, M.A. Social-ecological resilience through a biocultural lens: A participatory methodology to support global targets and local priorities (2020) Ecology and Society, 25 (3), art. no. 8, p. 1. DOI: 10.5751/ES-11621-250308

60. Xiong, Y., Sui, X., Ahmed, S., Wang, Z., Long, C. Ethnobotany and diversity of medicinal plants used by the Buyi in eastern Yunnan, China (2020) Plant Diversity, 42 (6), pp. 401-414. DOI: 10.1016/j.pld.2020.09.004

61. Rocha-Arriaga, C., Espinal-Centeno, A., Martinez-Sánchez, S., Caballero-Pérez, J., Alcaraz, L.D., Cruz-Ramírez, A. Deep microbial community profiling along the fermentation process of pulque, a biocultural resource of Mexico (2020) Microbiological Research, 241, art. no. 126593. DOI: 10.1016/j.micres.2020.126593

62. Thornton, S.A., Setiana, E., Yoyo, K., Dudin, Yulintine, Harrison, M.E., Page, S.E., Upton, C. Towards biocultural approaches to peatland conservation: The case for fish and livelihoods in Indonesia (2020) Environmental Science and Policy, 114, pp. 341-351. DOI: 10.1016/j.envsci.2020.08.018

63. Grey, S., Kuokkanen, R. Indigenous governance of cultural heritage: searching for alternatives to co-management (2020) International Journal of Heritage Studies, 26 (10), pp. 919-941. DOI: 10.1080/13527258.2019.1703202

64. Azul, D., Hancock, A.B. Who or what has the capacity to influence voice production? Development of a transdisciplinary theoretical approach to clinical practice addressing voice and the communication of speaker socio-cultural positioning (2020) International Journal of Speech-Language Pathology, 22 (5), pp. 559-570. DOI: 10.1080/17549507.2019.1709544

65. Hinton, D.E., Reis, R., de Jong, J. Ghost Encounters Among Traumatized Cambodian Refugees: Severity, Relationship to PTSD, and Phenomenology (2020) Culture, Medicine and Psychiatry, 44 (3), pp. 333-359. DOI: 10.1007/s11013-019-09661-6

66. McMillen, H.L., Campbell, L.K., Svendsen, E.S., Kealiikanakaoleohaililani, K., Francisco, K.S., Giardina, C.P. Biocultural stewardship, indigenous and local ecological knowledge, and the urban crucible (2020) Ecology and Society, 25 (2), art. no. 9, pp. 1-14. DOI: 10.5751/ES-11386-250209

67. Castiñeira Latorre, E., Canavero, A., Arim, M. Ethnobotanical Knowledge Complexity in a Conservation Area of Northern Uruguay: Interlocutors-Medicinal Plant Network and the Structural Patterns of Interaction (2020) Economic Botany, 74 (2), pp. 195-206. DOI: 10.1007/s12231-020-09491-3

68. Toyne, J.M., Murphy, M.S., Klaus, H.D. An introduction to advances in Andean South American paleopathology (2020) International Journal of Paleopathology, 29, pp. 1-15. DOI: 10.1016/j.ijpp.2019.09.001

69. Singh, A., Dubey, R.K., Bundela, A.K., Abhilash, P.C. The trilogy of wild crops, traditional agronomic practices, and un-sustainable development goals (2020) Agronomy, 10 (5), art. no. 648. DOI: 10.3390/agronomy10050648

70. Manzanero-Medina, G.I., Vásquez-Dávila, M.A., Lustre-Sánchez, H., Pérez-Herrera, A. Ethnobotany of food plants (quelites) sold in two traditional markets of Oaxaca, Mexico (2020) South African Journal of Botany, 130, pp. 215-223. DOI: 10.1016/j.sajb.2020.01.002

71. Landry Yuan, F., Ballullaya, U.P., Roshnath, R., Bonebrake, T.C., Sinu, P.A. Sacred groves and serpent-gods moderate human–snake relations (2020) People and Nature, 2 (1), pp. 111-122. DOI: 10.1002/pan3.10059

72. Stutz, A.J. The middle-upper paleolithic transition: A long-term biocultural effect of anatomically modern human dispersal (2020) Vertebrate Paleobiology and Paleoanthropology, pp. 157-186. DOI: 10.1007/978-3-030-46126-3_9

73. Álvarez-Ríos, G.D., Figueredo-Urbina, C.J., Casas, A. Physical, chemical, and microbiological characteristics of pulque: Management of a fermented beverage in Michoacán, Mexico (2020) Foods, 9 (3), art. no. 361. DOI: 10.3390/foods9030361

74. Thompson, K.-L., Hill, C., Ojeda, J., Ban, N.C., Picard, C.R. Indigenous food harvesting as social–ecological monitoring: A case study with the Gitga'at First Nation (2020) People and Nature, 2 (4), pp. 1085-1099. DOI: 10.1002/pan3.10135

75. Plieninger, T., Quintas-Soriano, C., Torralba, M., Mohammadi Samani, K., Shakeri, Z. Social dynamics of values, taboos and perceived threats around sacred groves in Kurdistan, Iran (2020) People and Nature, 2 (4), pp. 1237-1250. DOI: 10.1002/pan3.10158

76. Coe, M.A., Gaoue, O.G. Cultural keystone species revisited: are we asking the right questions? (2020) Journal of Ethnobiology and Ethnomedicine, 16 (1), art. no. 70. DOI: 10.1186/s13002-020-00422-z

77. Bond, M.O., Gaoue, O.G. Prestige and homophily predict network structure for social learning of medicinal plant knowledge (2020) PLoS ONE, 15 (10 October), art. no. e0239345. DOI: 10.1371/journal.pone.0239345

78. Jin, C., Zheng, M., Huang, L., Qian, S., Jim, C.Y., Lin, D., Zhao, L., Minor, J., Coggins, C., Chen, B., Zhang, J., Yang, Y. Co-existence between humans and nature: Heritage trees in China's yangtze River region (2020) Urban Forestry and Urban Greening, 54, art. no. 126748. DOI: 10.1016/j.ufug.2020.126748

79. Faast, R., Clarke, P.A., Taylor, G.S., Salagaras, R.L., Weinstein, P. Indigenous Use of Lerps in Australia: So Much More Than a Sweet Treat (2020) Journal of Ethnobiology, 40 (3), pp. 328-347. DOI: 10.2993/0278-0771-40.3.328

80. Oloriz, C., Parlee, B. Towards biocultural conservation: Local and indigenous knowledge, cultural values and governance of the White Sturgeon (Canada) (2020) Sustainability (Switzerland), 12 (18), art. no. 7320. DOI: 10.3390/SU12187320

81. Parra-Saldívar, A., Abades, S., Celis-Diez, J.L., Gelcich, S. Exploring perceived well-being from urban parks: Insights from a megacity in latin America (2020) Sustainability (Switzerland), 12 (18), art. no. 7586. DOI: 10.3390/su12187586

82. Valencia, M.R. The practice of co-production through biocultural design: A case study among the bribri people of Costa Rica and Panama (2020) Sustainability (Switzerland), 12 (17), art. no. 7120. DOI: 10.3390/su12177120

83. Pradhan, A., Ormsby, A.A. Biocultural conservation in the sacred forests of Odisha, India (2020) Environmental Conservation, 47 (3), pp. 190-196. DOI: 10.1017/S0376892920000181

84. Russell, S., Ens, E., Rangers, N.Y. 'We don't want to drink that water': Cross-cultural indicators of billabong water quality in remote Indigenous Australia (2020) Marine and Freshwater Research, 71 (10), pp. 1221-1233. DOI: 10.1071/MF19305

85. Wiley, A.S. Continuity and change in biocultural anthropology (2020) American Journal of Human Biology, 32 (4), art. no. e23464. DOI: 10.1002/ajhb.23464

86. Hicks, K. The role of biocultural approaches in assessing interventions for maternal weight and gestational weight gain (2020) American Journal of Human Biology, 32 (4), art. no. e23310. DOI: 10.1002/ajhb.23310

87. Winkel, T., Núñez-Carrasco, L., Cruz, P.J., Egan, N., Sáez-Tonacca, L., Cubillos-Celis, P., Poblete-Olivera, C., Zavalla-Nanco, N., Miño-Baes, B., Viedma-Araya, M.-P. Mobilising common biocultural heritage for the socioeconomic inclusion of small farmers: panarchy of two case studies on quinoa in Chile and Bolivia (2020) Agriculture and Human Values, 37 (2), pp. 433-447. DOI: 10.1007/s10460-019-09996-1

88. Gough, A. Symbiopolitics, Sustainability, and Science Studies: How to Engage With Alien Oceans (2020) Cultural Studies - Critical Methodologies, 20 (3), pp. 272-282. DOI: 10.1177/1532708619883314

89. Blom, D.E., Knudson, K.J. Paleopathology and children in the Andes: Local/situated biologies and future directions (2020) International Journal of Paleopathology, 29, pp. 65-75. DOI: 10.1016/j.ijpp.2019.08.004

90. Chamorro, M.F., Ladio, A. Native and exotic plants with edible fleshy fruits utilized in patagonia and their role as sources of local functional foods (2020) BMC Complementary Medicine and Therapies, 20 (1), art. no. 155. DOI: 10.1186/s12906-020-02952-1

91. Seare, A. Anabiosis and the liminal geographies of de/extinction (2020) Environmental Humanities, 12 (1), pp. 321-345. DOI: 10.1215/22011919-8142385

92. Tuuri, K., Koskela, O. Understanding Human–Technology Relations Within Technologization and Appification of Musicality (2020) Frontiers in Psychology, 11, art. no. 416. DOI: 10.3389/fpsyg.2020.00416

93. Zarazúa-Carbajal, M., Chávez-Gutiérrez, M., Romero-Bautista, Y., Rangel-Landa, S., Moreno-Calles, A.I., Ramos, L.F.A., Smith, S.E., Blancas, J., Del Val, E., Del Coro Arizmendi, M., Casas, A. Use and management of wild fauna by people of the Tehuacán-Cuicatlán Valley and surrounding areas, Mexico (2020) Journal of Ethnobiology and Ethnomedicine, 16 (1), art. no. 4. DOI: 10.1186/s13002-020-0354-8

94. Vilá, B., Arzamendia, Y. South American Camelids: their values and contributions to people (2020) Sustainability Science. DOI: 10.1007/s11625-020-00874-y

95. Rana, S., Ávila-García, D., Dib, V., Familia, L., Gerhardinger, L.C., Martin, E., Martins, P.I., Pompeu, J., Selomane, O., Tauli, J.I., Tran, D.H.T., Valle, M., von Below, J., Pereira, L.M. The voices of youth in envisioning positive futures for nature and people (2020) Ecosystems and People, 16 (1), pp. 326-344. DOI: 10.1080/26395916.2020.1821095

96. Puentes, J.P., Arenas, P.M., Hurrell, J.A. Medicinal and aromatic lamiaceae commercialized in the buenos aires metropolitan area, Argentina (2020) Bonplandia, 29 (1), pp. 5-20. DOI: 10.30972/BON.2914106

97. Aigo, J.D.C., Skewes, J.C., Bañales-Seguel, C., Riquelme Maulén, W., Molares, S., Morales, D., Ibarra, M.I., Guerra, D. WATERSCAPES IN WALLMAPU: LESSONS FROM MAPUCHE PERSPECTIVES (2020) Geographical Review. DOI: 10.1080/00167428.2020.1800410

98. Smith-Oka, V., Nissen, N.J., Wornhoff, R., Sheridan, S.G. “I Thought I Was Going to Die”: Examining Experiences of Childbirth Pain Through Bioarchaeological and Ethnographic Perspectives (2020) Bioarchaeology and Social Theory, pp. 149-176. DOI: 10.1007/978-3-030-32181-9_8

99. Clark, M.A., Bargielski, R., Reich, D. Adult paleopathology as an indicator of childhood social roles: A case study of Perthes disease in a native Ohio female (2020) International Journal of Osteoarchaeology, 30 (1), pp. 24-32. DOI: 10.1002/oa.2826

100. Pieroni, A., Sõukand, R., Bussmann, R.W. The Inextricable Link Between Food and Linguistic Diversity: Wild Food Plants among Diverse Minorities in Northeast Georgia, Caucasus (2020) Economic Botany, 74 (4), pp. 379-397. DOI: 10.1007/s12231-020-09510-3

101. Kazancı, C., Oruç, S., Mosulishvili, M. Medicinal ethnobotany of wild plants: a cross-cultural comparison around Georgia-Turkey border, the Western Lesser Caucasus (2020) Journal of Ethnobiology and Ethnomedicine, 16 (1), art. no. 71. DOI: 10.1186/s13002-020-00415-y

102. Marklein, K.E. East of Rome: Exploring potential impacts of Roman imperialism on Northeastern Mediterranean populations through a bioarchaeological perspective (2020) Journal of Archaeological Science: Reports, 34, art. no. 102590. DOI: 10.1016/j.jasrep.2020.102590

103. Rivera López, F., Wickson, F., Helen Hausner, V. Bridging different perspectives for biocultural conservation: art-based participatory research on native maize conservation in two indigenous farming communities in Oaxaca, Mexico (2020) Environment, Development and Sustainability, 22 (8), pp. 7427-7451. DOI: 10.1007/s10668-019-00530-1

104. Marquet, P.A., Castilla, J.C., Gaxiola, A., Hucke-Gaete, R., Pena-Vega, A. Indigenous rights to Patagonia's Guafo island (2020) Science, 370 (6517), pp. 669-670. DOI: 10.1126/science.abf1962

105. Pollegioni, P., Lungo, S.D., Müller, R., Woeste, K.E., Chiocchini, F., Clark, J., Hemery, G.E., Mapelli, S., Villani, F., Malvolti, M.E., Mattioni, C. Biocultural diversity of common walnut (Juglans regia L.) and sweet chestnut (Castanea sativa Mill.) across Eurasia (2020) Ecology and Evolution, 10 (20), pp. 11192-11216. DOI: 10.1002/ece3.6761

106. Barbosa, E.C., Cruz, R.S., dos Santos, G.M.C., da Silva, R.R.V., de Albuquerque, U.P., de Medeiros, P.M. Use Categories and Local Perception of Decline in Plant Populations: a Case Study of Woody Medicinal Plants in Northeastern Brazil (2020) Economic Botany, 74 (3), pp. 356-362. DOI: 10.1007/s12231-020-09502-3

107. Houghton, L.C., Troisi, R., Sommer, M., Katki, H.A., Booth, M., Choudhury, O.A., Hampshire, K.R. "I'm not a freshi": Culture shock, puberty and growing up as British-Bangladeshi girls (2020) Social Science and Medicine, 258, art. no. 113058. DOI: 10.1016/j.socscimed.2020.113058

108. Quinn, E.A., Childs, G. Trekking and toddlers: Towards a biocultural analysis of growth among infants and young children in a rural region of the Himalayas (2020) American Journal of Human Biology, 32 (4), art. no. e23452. DOI: 10.1002/ajhb.23452

109. Jennings, J.A. Hired helpers at the nest: The association between life-cycle servants and net fertility in North Orkney, 1851–1911(2020). American Journal of Physical Anthropology, 172 (3), pp. 412-422. DOI: 10.1002/ajpa.24040

110. Gibbon, V.E., Davies, B. Holocene Khoesan health: a biocultural analysis of cranial pathology and trauma(2020) International Journal of Osteoarchaeology, 30 (3), pp. 287-296. DOI: 10.1002/oa.2854

111. Gutiérrez-García, L., Labrador-Moreno, J., Blanco-Salas, J., Monago-Lozano, F.J., Ruiz-Téllez, T. Food identities, biocultural knowledge and gender differences in the protected area "sierra grande de hornachos" (Extremadura, spain)(2020) International Journal of Environmental Research and Public Health, 17 (7), art. no. 2283. DOI: 10.3390/ijerph17072283

112. Herrera-Cabrera, B.E., Campos Contreras, J.E., Macías-Cuéllar, H., Delgado-Alvarado, A., Salazar-Rojas, V.M. Beyond the traditional home garden: a circa situm conservation experience of Laelia anceps subsp.

dawsonii f. chilapensis Soto-Arenas (Orchidaceae)(2020) Environment, Development and Sustainability, 22 (3), pp. 1913-1927. DOI: 10.1007/s10668-018-0270-4

113. Stutz, A.J. A niche of their own: population dynamics, niche diversification, and biopolitics in the recent biocultural evolution of hunter-gatherers (2020) Journal of Anthropological Archaeology, 57, art. no. 101120. DOI: 10.1016/j.jaa.2019.101120

114. Doumecq, M.B., Arenas, P.M., Hurrell, J.A. Ethnobotany of the fuel species commercialized in the Rio de la Plata riverside, Buenos Aires, Argentina [Etnobotánica de las especies combustibles comercializadas en la ribera platense, buenos aires, argentina](2020) Ethnobotany Research and Applications, 19, art. no. 3. DOI: 10.32859/era.19.03.1-27

115. Medina, Y., Massardo, F., Rozzi, R. EDUCATION, ECOTOURISM, AND BIOCULTURAL CONSERVATION IN THE MINIATURE FORESTS OF CAPE HORN [EDUCACIÓN, ECOTURISMO Y CONSERVACIÓN BIOCULTURAL EN LOS BOSQUES EN MINIATURA DEL CABO DE HORNOS] (2020) Magallania, 48 (2), pp. 183-211. DOI: 10.4067/S0718-22442020000200183

116. Sheridan, S.G. Pious Pain: Repetitive Motion Disorders from Excessive Genuflection at a Byzantine Jerusalem Monastery (2020) Bioarchaeology and Social Theory, pp. 81-117. DOI: 10.1007/978-3-030-32181-9_5

117. Torres-Mazuera, G., Vides Borrell, E., Rivera, F. Democratizar la bioseguridad en territorios con diversidad biocultural: la apuesta por una alianza de saberes en México (2020) Journal of Political Ecology, 27 (1), pp. 1036-1051. DOI: 10.2458/V27I1.23211

118. Gevorkyan, S., Segovia, C.A. Paul and the Plea for Contingency in Contemporary Philosophy: A Philosophical and Anthropological Critique (2020) Open Philosophy, 3 (1), pp. 625-656. DOI: 10.1515/opphil-2020-0142

119. Palmquist, A. Cooperative Lactation and the Mother-Infant Nexus (2020) Bioarchaeology and Social Theory, pp. 125-142. DOI: 10.1007/978-3-030-27393-4_7

120. Cheyney, M., Davis-Floyd, R. Birth and the Big Bad Wolf: Biocultural Evolution and Human Childbirth, Part 2 (2020) International Journal of Childbirth, 10 (2), pp. 66-78. DOI: 10.1891/IJCBIRTH-D-19-00029

121. Coombes, B. Nature's rights as Indigenous rights? Mis/recognition through personhood for Te Urewera [Les droits de la nature comme droits autochtones? Mal/reconnaissance par le statut de personne pour Te Urewera] (2020) Espace-Populations-Societes, 2020 (1-2. DOI: 10.4000/EPS.9857

122. Califano, L.M. Perception and management of landscape and vegetable resources by trashumants cattle 9-farmers from Iruya (Salta, Argentina) (2020) Bonplandia, 29 (1), pp. 101-118. DOI: 10.30972/BON.2914112

123. Cruz, R.S., de Medeiros, P.M., Ferreira, W.S., Jr., da Silva, R.R.V. Factors that influence human behavior in fuelwood use and their implications for biocultural conservation (2020) Ethnobiology and Conservation, 9, pp. 1-13. DOI: 10.15451/EC2020-07-9.31-1-13

124. Vidal, O., Brusca, R.C. Mexico's biocultural diversity in peril [La diversidad biocultural de México en peligro](2020) Revista de Biologia Tropical, 68 (2), pp. 669-691.

125. Lee, K.-C., Karimova, P.G., Yan, S.-Y., Li, Y.-S. Resilience assessment workshops: A biocultural approach to conservation management of a rural landscape in Taiwan (2020) Sustainability (Switzerland), 12 (1), art. no. 5559. DOI: 10.3390/SU12010408

126. Puentes, J.P., Robles, G. Fresh fruits and derived products commercialized in the metropolitan area of Buenos Aires, Argentina [Frutas frescas y productos derivados comercializados en el área metropolitana

de Buenos Aires, Argentina] (2020) Boletin Latinoamericano y del Caribe de Plantas Medicinales y Aromaticas, 19 (1), pp. 77-125.

127. Alvarado, A.M., García-Trejo, F., Cardador-Martínez, A., Magallán-Hernández, F. Clinopodium mexicanum: Potential and difficulties for the sustainable use of a Mexican medicinal plant [Clinopodium mexicanum: Potencial y dificultades para el uso de una planta medicinal Mexicana] (2020) Boletin Latinoamericano y del Caribe de Plantas Medicinales y Aromaticas, 19 (2), pp. 149-160.

128. Zapico, F.L., DIzon, J.T., Borromeo, T.H., McNally, K.L., Fernando, E.S., Hernandez, J.E. Genetic erosion in traditional rice agro-ecosystems in Southern Philippines: Drivers and consequences (2020) Plant Genetic Resources: Characterisation and Utilisation, 18 (1), pp. 1-10. DOI: 10.1017/S1479262119000406

129. García Flores, A. Ethnoecological study of the birds of Coatetelco, Morelos, Mexico [Estudio etnoecológico de las aves de Coatetelco, Morelos, México](2020) Ecosistemas, 29 (3), art. no. 1942. DOI: 10.7818/ECOS.1942

130. Argumedo, A., Song, Y., Khoury, C.K., Hunter, D., Dempewolf, H. Support Indigenous food system biocultural diversity (2020) The Lancet Planetary Health, 4 (12), p. e554. DOI: 10.1016/S2542-5196(20)30243-6

131. Hubbard, A.R., Monnig, L.A. Using Anthropological Principles to Transform the Teaching of Human “Difference” and Genetic Variation in College Classrooms (2020) Science and Education, 29 (6), pp. 1541-1565. DOI: 10.1007/s11191-020-00164-0

132. Cheng, Z., Luo, B., Fang, Q., Long, C. Ethnobotanical study on plants used for traditional beekeeping by Dulong people in Yunnan, China (2020) Journal of Ethnobiology and Ethnomedicine, 16 (1), art. no. 61. DOI: 10.1186/s13002-020-00414-z

133. Reyes-Valdés, M.H., Kantartzi, S.K. An information theory approach to biocultural complexity (2020) Scientific Reports, 10 (1), art. no. 7203. DOI: 10.1038/s41598-020-64260-5

134. Singh, S.J., Fischer-Kowalski, M., Chertow, M. Introduction: The metabolism of islands (2020) Sustainability (Switzerland), 12 (22), art. no. 9516, pp. 1-8. DOI: 10.3390/su12229516

135. Buchanan, A. Précis: Our moral fate: Evolution and the escape from tribalism (2020) Analyse und Kritik, 42 (2), pp. 443-447. DOI: 10.1515/auk-2020-0018

136. Narchi, N.E., Vanderplank, S.E., Medina-Rodríguez, J., Alfaro-Mercado, E. Environmental Violence and the Socio-environmental (de)Evolution of a Landscape in the San Quintín Valley (2020) Latin American Perspectives, 47 (6), pp. 103-118. DOI: 10.1177/0094582X20951774

137. Albuquerque, U.P., Ludwig, D., Feitosa, I.S., de Moura, J.M.B., de Medeiros, P.M., Gonçalves, P.H.S., da Silva, R.H., da Silva, T.C., Gonçalves-Souza, T., Ferreira Júnior, W.S. Addressing Social-Ecological Systems across Temporal and Spatial Scales: a Conceptual Synthesis for Ethnobiology (2020) Human Ecology, 48 (5), pp. 557-571. DOI:10.1007/s10745-020-00189-7

138. Califano, L.M. Grazing management: Knowledge and practices of fodder species management in transhumant livestock farming in Iruya (Salta, Argentina) [Gestión del pastoreo: Conocimientos y prácticas de manejo de las especies forrajeras en la ganadería trashumante de Iruya (Salta, Argentina)] (2020) Boletin de la Sociedad Argentina de Botanica, 55 (3), pp. 493-513. DOI: 10.31055/1851.2372.v55.n3.28119

139. Magalhães, B.M., Mays, S., Santos, A.L. A new approach to recording nasal fracture in skeletonized individuals(2020) International Journal of Paleopathology, 30, pp. 105-109. DOI: 10.1016/j.ijpp.2020.04.003

140. Pietrobelli, A., Mariotti, V., Fusari, S., Gasparini, A., Bettuzzi, M., Morigi, M.P., Belcastro, M.G. Syphilis in an Italian medieval jewish community: A bioarchaeological and cultural perspective (2020) International Journal of Paleopathology, 30, pp. 85-97. DOI: 10.1016/j.ijpp.2020.06.001

141. Venkatesan, S., Peter, A.M. Feminine famishment: Graphic medicine and anorexia nervosa (2020) Health (United Kingdom), 24 (5), pp. 518-534. DOI: 10.1177/1363459318817915

142. Garavito-Bermúdez, D. Biocultural learning–beyond ecological knowledge transfer (2020) Journal of Environmental Planning and Management, 63 (10), pp. 1791-1810. DOI: 10.1080/09640568.2019.1688651

143. Cheverko, C.M., Prince-Buitenhuys, J.R., Hubbe, M. Theoretical approaches in bioarchaeology (2020) Theoretical Approaches in Bioarchaeology, pp. 1-210. DOI: 10.4324/9780429262340

144. Py-Saragaglia, V., Bal, M.-C., Brun, C., Buscaino, S., Guillerme, S., Philippe, M., Saulnier, M., Tămaş, C.G., Burri, S., Calastrenc, C., Poirier, N., Danu, M., Ioan, A., de Vleeschouwer, F., Brin, A., Ladet, S., Larrieu, L., Le Roux, G., Mîndrescu, M., Petras, A., Roy, M. Knowledge and conservation of old-growth forests: A key issue to face global changes: The case study of Strâmbu-Băiuț - Maramureş (Eastern Carpathians, Romania) (2020) Quaderni Storici, 55 (2), pp. 369-404. DOI: 10.1408/99412

145. Turek, J. Beer, Pottery, Society and Early European Identity (2020) Archaeologies, 16 (2), pp. 396-423. DOI: 10.1007/s11759-020-09406-7

146. Pyhälä, A. Resistance to 'development' amongst the Kogui of the Sierra Nevada de Santa Marta (2020) Indigenous Wellbeing and Enterprise: Self-Determination and Sustainable Economic Development, pp. 63-87. DOI: 10.4324/9780429329029-4

147. Rubio, M., Figueroa, F., Zambrano, L. Dissonant Views of Socioecological Problems: Local Perspectives and Conservation Policies in Xochimilco, Mexico (2020) Conservation and Society, 18 (3), pp. 207-219. DOI: 10.4103/cs.cs_19_72

148. Peterson, D., Hanazaki, N., Berkes, F. Do We All Speak the Same Language When Talking Conservation? Caiçąra Understandings of Conservation in their Landscape (2020) Conservation and Society, 18 (3), pp. 238-251. DOI: 10.4103/cs.cs_18_123

149. Cagüeñas, D., Orrego, M.I.G., Rasmussen, S. The atrato river and its guardians: Ecopolitical imagination for weaving new rights [El Atrato y sus guardianes: Imaginación ecopolítica para hilar nuevos derechos](2020) Revista Colombiana de Antropologia, 56 (2), pp. 169-196. DOI: 10.22380/2539472X.638

150. Leonard, W.R. Re-examining Biocultural Approaches in Human Biology (2020) American Journal of Human Biology, 32 (4), art. no. e23475. DOI: 10.1002/ajhb.23475

151. Romero-Bautista, Y.A., Moreno-Calles, A.I., Alvarado-Ramos, F., Reyes Castillo, M., Casas, A. Environmental interactions between people and birds in semiarid lands of the Zapotitlán Valley, Central Mexico(2020) Journal of Ethnobiology and Ethnomedicine, 16 (1), art. no. 32. DOI: 10.1186/s13002-020-00385-1

152. Cowell, C.R., Anderson, P.M.L., Annecke, W.A. Historic herbarium specimens as biocultural assets: An examination of herbarium specimens and their in situ plant communities of the Agulhas National Park, South Africa(2020) People and Nature, 2 (2), pp. 483-494. DOI: 10.1002/pan3.10087

153. Ibarguën, M.A., Beriain, J. Struggles on the futures of human nature. Sociological perspectives [Las disputas sobre los futuros de la naturaleza humana. Perspectivas sociológicas](2020) Empiria, (47), pp. 105-127. DOI: 10.5944/EMPIRIA.47.2020.27426

154. Temudo, M.P., Oom, D., Pereira, J.M. Bio-cultural fire regions of Guinea-Bissau: Analysis combining social research and satellite remote sensing (2020) Applied Geography, 118, art. no. 102203. DOI: 10.1016/j.apgeog.2020.102203

155. Veleski, S. A scientific turn in the genre of how-to fiction writing manuals? (2020) Evolutionary Studies in Imaginative Culture, 4 (1), pp. 91-104. DOI: 10.26613/esic.4.1.173

156. McClure, S. Living Unembodiment: Physicality and Body/Self Discontinuity Among African American Adolescent Girls (2020) Ethos, 48 (1), pp. 3-28. DOI: 10.1111/etho.12266

157. Falu, N. Ain't I Too a Mulher? Implications of Black Lesbians' Well-being, Self-care, and Gynecology in Brazil(2020) Journal of Latin American and Caribbean Anthropology, 25 (1), pp. 48-66. DOI: 10.1111/jlca.12464

158. Nabhan, G.P. Biocultural restoration of sacred sites, earth day, and restoration ecology's patron saint(2020) Ecological Restoration, 38 (1), pp. 54-55. DOI: 10.3368/er.38.1.54

159. James, G.D. Allostasis and Adaptation: Biocultural Processes Integrating Lifestyle, Life History, and Blood Pressure Variation (2020) American Anthropologist, 122 (1), pp. 51-64. DOI: 10.1111/aman.13366

160. Seyler, B.C., Gaoue, O.G., Tang, Y., Duffy, D.C., Aba, E. Collapse of orchid populations altered traditional knowledge and cultural valuation in Sichuan, China (2020) Anthropocene, 29, art. no. 100236. DOI: 10.1016/j.ancene.2020.100236

161. de Siqueira, J.I.A., Machado, T.J., Lemos, J.R. Bioculturalidade associada à extração e uso do azeite de uma etnovariedade de sesamum l. (pedaliaceae): Uma abordagem etnobotânica em uma comunidade rural no semiárido do piauí (nordeste do brasil) (2020) Ethnobotany Research and Applications, 19, art. no. 11. DOI: 10.32859/era.19.11.1-26

162. Hookway, E., Squires, K. A Biocultural Approach to Understanding the Presence of Children from Medieval Hospitals in England: What Can We Learn from Archaeological Investigations? (2020) Childhood in the Past, 13 (1), pp. 38-59. DOI: 10.1080/17585716.2020.1739281

163. Narváez-Elizondo, R.E., González-Elizondo, M., Castro-Castro, A., González-Elizondo, M.S., Tena-Flores, J.A., Chairez-Hernández, I. Comparison of traditional knowledge about edible plants among young southern tepehuans of Durango, Mexico [Comparación de conocimientos tradicionales sobre plantas comestibles entre jóvenes tepehuanes del sur de Durango, México](2020) Botanical Sciences, 1 (1). DOI: 10.17129/BOTSCI.2792

164. Barreto, M.L., Rendón, M.P. Biocultural indicators in environmental management projects. The case of meliponiculture in Yucatan [Indicadores bioculturales en proyectos de gestión ambiental. El caso de la meliponicultura en Yucatán] (2020) Polis (Italy), 19 (57), pp. 52-72. DOI: 10.32735/S0718-6568/2020-N57-1564

165. Stoenescu, C. The biocultural ethics and the homogeneous sustainable society (2020) Public Reason, 12 (1), pp. 7-17.

166. Gimenez, T.V. From climate justice to ecological justice: The rights of nature [De la justicia climÁtica a la justicia ecolÓgica: Los derechos de la naturaleza] (2020) Revista Catalana de Dret Ambiental, 11 (2). DOI: 10.17345/rcda2842

167. Higuero Pliego, A. Reflexionando acerca del poder de las narrativas sociales frente a los discursos científicos en tiempos de pandemia: Del VIH al COVID (2020) Revista Espanola de Antropologia Fisica, 42, pp. 71-74.

168. Lende, D.H. The Purposeful Pain of Drug Addiction: A Biocultural Approach (2020) Bioarchaeology and Social Theory, pp. 177-193. DOI: 10.1007/978-3-030-32181-9_9

169. Halcrow, S., Gowland, R. Concluding Thoughts: Small Beginnings, Significant Outcomes (2020) Bioarchaeology and Social Theory, pp. 275-277. DOI: 10.1007/978-3-030-27393-4_15

170. Turner, B.L., Klaus, H.D. The Lambayeque Biohistory Project: Contexts and Analysis (2020) Bioarchaeology and Social Theory, pp. 85-111. DOI: 10.1007/978-3-030-42614-9_6

171. Turner, B.L., Klaus, H.D. Theorizing Food and Power in the Ancient Andes (2020) Bioarchaeology and Social Theory, pp. 11-28. DOI: 10.1007/978-3-030-42614-9_2

172. Reedy, S. Conclusion (2020) Bioarchaeology and Social Theory, pp. 277-282. DOI: 10.1007/978-3-030-46440-0_12

173. Tung, T.A. Performing Identity and Revealing Structures of Violence Through Purposeful Pain (2020) Bioarchaeology and Social Theory, pp. 253-264. DOI: 10.1007/978-3-030-32181-9_13

174. Stone, P.K. Female Beauty, Bodies, Binding, and the Bioarchaeology of Structural Violence in the Industrial Era Through the Lens of Critical White Feminism (2020) Bioarchaeology and Social Theory, pp. 13-30. DOI: 10.1007/978-3-030-46440-0_2

175. Robbins Schug, G. Touching the Surface: Biological, Behavioural, and Emotional Aspects of Plagiocephaly at Harappa (2020) Bioarchaeology and Social Theory, pp. 235-256. DOI: 10.1007/978-3-030-27393-4_13

176. Turner, B.L., Klaus, H.D. Results: Paleopathological and Stable Isotope Findings (2020) Bioarchaeology and Social Theory, pp. 113-156. DOI: 10.1007/978-3-030-42614-9_7

177. Sheridan, S.G., Gregoricka, L.A. A Bioarchaeology of Purposeful Pain (2020) Bioarchaeology and Social Theory, pp. 1-17. DOI: 10.1007/978-3-030-32181-9_1

178. Svensson, E., Haas, J., Eckstein, R.L. Integrating Nature and Heritage in the Boreal Forests of Scandinavia? Exploration of a Low-Budget Method (2020) Landscapes (United Kingdom), 21 (1), pp. 72-92. DOI: 10.1080/14662035.2020.1905202

179. López Barreto, M., Pinkus Rendón, M. Biocultural indicators in environmental management projects. The case of meliponiculture in Yucatan [Indicadores bioculturales en proyectos de gestión ambiental. El caso de la meliponicultura en Yucatán] (2020) Polis (Italy), (57), pp. 52-72. DOI: 10.32735/s0718-6568/2021-n57-1564

180. Staiano-Ross, K. Propositions for a biocultural semiotics [Bio-kultuurisemiootika teesid] [Предложения по биокультурной семиотике] (2020) Sign Systems Studies, 48 (2-4), pp. 450-482. DOI: 10.12697/SSS.2020.48.2-4.12

181. Craighead, K.A., Yacelga, M. Indigenous peoples' displacement and jaguar survival in a warming planet (2020) Global Sustainability, art. no. e7. DOI: 10.1017/sus.2021.6

182. Martínez, M.A.C., Morfín, L.M., Espinoza, P.O.H. The mortality of children under five years of age in the parish of El Sagrario, Zacatecas (Mexico) between 1835-1845 [La mortalidad de los menores de cinco anõs en la parroquia del Sagrario, Zacatecas (México) entre 1835-1845] (2020) Revista de Demografia Historica, 38 (2), pp. 57-81.

183. Last, C. Atechnogenesis and Technocultural Evolution (2020) World-Systems Evolution and Global Futures, pp. 165-188. DOI: 10.1007/978-3-030-46966-5_9

184. Last, C. Biocultural Theory of Human Reproduction (2020) World-Systems Evolution and Global Futures, pp. 151-164. DOI: 10.1007/978-3-030-46966-5_8

185. Last, C. Control Dynamics of Human Metasystems (2020) World-Systems Evolution and Global Futures, pp. 83-96. DOI: 10.1007/978-3-030-46966-5_5

186. Valadez-Blanco, O., Morales-Zaragoza, N.A., González-García, D.N. Catalyzing biocultural and social changes about cancer. Design of transdisciplinary actions against the pandemic [Catalizando cambios sociales y bioculturales sobre el cáncer. Diseño de acciones transdisciplinarias contra la pandemia] (2020) Revista de Salud Publica, 22 (3), pp. 1-10. DOI: 10.15446/RSAP.V22N3.87147

187. de Siqueira, J.I.A., Medeiros, M.F.T., de Senna-Valle, L. A methodological proposal for the use of short stories regarding the biocultural memory as a pedagogical tool (2020) Ethnobotany Research and Applications, 20, art. no. 32, pp. 1-9. DOI: 10.32859/era.20.32.1-9

188. Krupar, S., Ehlers, N. Biocultures: a critical approach to mundane biomedical governance (2020) Culture, Theory and Critique, 61 (4), pp. 440-456. DOI: 10.1080/14735784.2020.1857810

189. Arı, Y. Protecting biocultural diversity at Kazdaği national park, Balikesir, Turkey: The role of sacred natural sites (2020) Human Geographies, 14 (2), pp. 215-238. DOI: 10.5719/hgeo.2020.142.3

190. Santiago, C.M., Acuña, N.F., Luks, S.K., Ibarra, J.T. Local knowledge in montane homegardens in the southern Andes: A refuge of Mapuche Pewenche biocultural memory [Saberes locales en huertas de montaña del sur de los andes: Un refugio de memoria biocultural mapuche pewenche] (2020) Pirineos, 175, art. no. e060. DOI: 10.3989/PIRINEOS.2020.175010

191. Fentaw, E., Dagne, K., Wondimu, T., Demissew, S., Bjorå, C.S., Grace, O.M. Uses and perceived sustainability of Aloe L. (Asphodelaceae) in the central and northern Highlands of Ethiopia (2020) South African Journal of Botany. DOI: 10.1016/j.sajb.2020.11.001

192. Portus, R. An ecological whodunit: The story of colony collapse disorder (2020) Society and Animals. DOI: 10.1163/15685306-BJA10026

193. Jouault, S. ¿Dónde están los mayas ? Community-based tourism as an identity claim in Yucatan [¿Dónde están los mayas ? Le tourisme communautaire comme revendication identitaire dans le Yucatán] (2020) Espace-Populations-Societes, 2020 (1-2). DOI: 10.4000/EPS.9963

194. Doumecq, M.B., Petrucci, N.S., Stampella, P.C. When knowledges do not talk. Practices in conflict in the Parque Costero del Sur (Province of Buenos Aires) (2020) Bonplandia, 29 (1), pp. 57-70. DOI: 10.30972/BON.2914109

195. Erazo, V.A., González, A.E., García, N., Bernal, R., Raz, L., Galeano, G. Use, management and local ecological knowledge of Sabal mauritiiformis in the Colombian Caribbean (2020) Ethnobiology and Conservation, 9, art. no. 24. DOI: 10.15451/EC2020-05-9.15-1-24

196. Kinaston, R.L., Koesbardiati, T., Suriyanto, R.A., Buckley, H.R., Halcrow, S.E., Foster, A., Simanjuntak, T., Bedford, S., Murti, D.B., Putri, R.S., Galipaud, J.-C. Ritual tooth ablation and the Austronesian expansion: Evidence from eastern Indonesia and the Pacific Islands (2020) Journal of Island and Coastal Archaeology. DOI: 10.1080/15564894.2020.1754971

197. de Lira Azevêdo, E., Drumond, M.A., Alves, R.R.N., Dias, T.L.P., Molozzi, J. Evaluating conservation threats to reservoirs in the semiarid region of Brazil using the perception of residents (2020) Ethnobiology and Conservation, 9, pp. 1-15. DOI: 10.15451/EC2020-02-9.04-1-15

198. de Siqueira, J.I.A., Vieira, I.R., Chaves, E.M.F., Diago, O.L.S., Lemos, J.R. Biocultural behavior and traditional practices on the use of species of euphorbiaceae in rural home gardens of the semiarid region of piauí state (NE, Brazil) [Comportamiento biocultural y prácticas tradicionales sobre el uso de especies de euphorbiaceae en huertos familiares en región semiárida del estado de piauí (NE, Brasil)] (2020) Caldasia, 42 (1), pp. 70-84. DOI: 10.15446/caldasia.v42n1.76202

199. Vera, L., García, S. International Recognition of the Biocultural Protection in Dryland Regions: The World Heritage Property in the Tehuacán-Cuicatlán Biosphere Reserve (2020) Springer Climate, pp. 215-226. DOI: 10.1007/978-3-030-22464-6_13
